# Transcriptome Profile of the Response of Paracoccidioides spp. to a Camphene Thiosemicarbazide Derivative

**DOI:** 10.1371/journal.pone.0130703

**Published:** 2015-06-26

**Authors:** Lívia do Carmo Silva, Diana Patrícia Tamayo Ossa, Symone Vitoriano da Conceição Castro, Ludmila Bringel Pires, Cecília Maria Alves de Oliveira, Cleuza Conceição da Silva, Narcimário Pereira Coelho, Alexandre Melo Bailão, Juliana Alves Parente-Rocha, Célia Maria de Almeida Soares, Orville Hernández Ruiz, Juan G. McEwen Ochoa, Maristela Pereira

**Affiliations:** 1 Laboratório de Biologia Molecular, Instituto de Patologia Tropical e Saúde Pública Universidade Federal de Goiás, Goiânia, Brazil; 2 Unidad de Biología Celular y Molecular, Corporación para Investigaciones Biológicas (CIB) and Facultad de Medicina Universidad de Antioquia, Medellín, Colombia; 3 Laboratório de Produtos Naturais, Instituto de Química, Universidade Federal de Goiás, Goiânia, Brazil; 4 Laboratório de Fitoquímica e Síntese Orgânica, Departamento de Química, Universidade Estadual de Maringá, Paraná, Brazil; Instituto de Salud Carlos III, SPAIN

## Abstract

Paracoccidioidomycosis (PCM) is a systemic granulomatous human mycosis caused by fungi of the genus *Paracoccidioides*, which is geographically restricted to Latin America. Inhalation of spores, the infectious particles of the fungus, is a common route of infection. The PCM treatment of choice is azoles such as itraconazole, but sulfonamides and amphotericin B are used in some cases despite their toxicity to mammalian cells. The current availability of treatments highlights the need to identify and characterize novel targets for antifungal treatment of PCM as well as the need to search for new antifungal compounds obtained from natural sources or by chemical synthesis. To this end, we evaluated the antifungal activity of a camphene thiosemicarbazide derivative (TSC-C) compound on *Paracoccidioides* yeast. To determine the response of *Paracoccidioides spp*. to TSC-C, we analyzed the transcriptional profile of the fungus after 8 h of contact with the compound. The results demonstrate that *Paracoccidioides lutzii* induced the expression of genes related to metabolism; cell cycle and DNA processing; biogenesis of cellular components; cell transduction/signal; cell rescue, defense and virulence; cellular transport, transport facilities and transport routes; energy; protein synthesis; protein fate; transcription; and other proteins without classification. Additionally, we observed intensely inhibited genes related to protein synthesis. Analysis by fluorescence microscopy and flow cytometry revealed that the compound induced the production of reactive oxygen species. Using an isolate with down-regulated *SOD1* gene expression (*SOD1*-aRNA), we sought to determine the function of this gene in the defense of *Paracoccidioides* yeast cells against the compound. Mutant cells were more susceptible to TSC-C, demonstrating the importance of this gene in response to the compound. The results presented herein suggest that TSC-C is a promising candidate for PCM treatment.

## Introduction

Paracoccidioidomycosis (PCM) is a systemic mycosis geographically restricted to Latin America caused by thermodimorphic fungi of the genus *Paracoccidioides*. The fungi usually infect the host through the respiratory tract by inhalation of conidia, which are the infectious propagules found in the environment. In the lungs, these propagules differentiate into the pathogenic form in a temperature-dependent manner, corresponding to the yeast phase of the fungus, and spreads to other organs through lymphohematogenous dissemination. Because this mycosis affects mainly rural males of working age between the ages of 30 and 50 years, the disease has socioeconomic repercussions due to its potential to debilitate. In addition to the lungs, PCM frequently compromises the mucous membranes, lymph nodes, liver, spleen and bone marrow [1,2].

Treating PCM remains a challenge due to the toxicity of the antifungals commonly used to treat this mycosis—sulfonamides, azoles and polyenes [3,4]. Additionally, despite the use of antifungals, individuals with PCM have persistent latent foci, which slow down treatment and may extend it over months or years depending on the severity of the disease and the site of injury [5,6,7]. Thus, the need to research and develop new therapeutic approaches is increasingly evident. With this aim, our group has invested effort into identifying and characterizing novel targets for antifungal drugs against *Paracoccidioides spp*. [8–16] and searching for new antifungal compounds obtained from natural sources or their synthetic derivatives [17,18,19].

The monoterpenoids are the components of essential oils, which are produced in large quantities by plants. These molecules are significant due to their therapeutic potential, low cost as well as the commercial availability, being used as starting material for synthesis of bioactive compounds [20,21]. Following this approach a series of thiosemicarbazides and thiosemicarbazones deriving from bisabolol, kaurenoic acid, limonene and camphene were synthetized by our research group [22,23,24]. Among them, the tiosemicarbazide camphene derivative (TSC-C) showed remarkable antifungal activity. The previous study showed that TSC-C inhibited the growth of *Trichophyton mentagrophytes* by damaging the cell wall structure or interfering with its formation during the process of cell division, growth or morphogenesis [24]. Based on these results, we elected TSC-C to study its activity and mode of action on *Paracoccidioides brasiliensis*.

We constructed a cDNA library to obtain expressed sequences tags (ESTs) from *P*. *lutzii* in response to TSC-C with the ultimate aim to identify the likely mode of action of the compound in the fungus. We performed assays to confirm the transcriptome data to *P*. *lutzii* and *Paracoccidioides brasiliensis*, such as quantitative real-time PCR (qRT-PCR), fluorescence microscopy, DNA fragmentation, cell cycle analysis by flow cytometry and enzymatic assays.

## Materials and Methods

### General procedure for the preparation of compounds

The TSC-C was prepared as described by Yamaguchi [24].

### Microorganism and cell culture

The *P*. *lutzii* ATCC MYA 826 and *P*. *brasiliensis* ATCC 60855 strains were used in the assays. Yeast cells were maintained in Fava-Netto liquid medium [25] for 3 days. The cells were then transferred and grown overnight in McVeigh Morton (MMcM) liquid medium overnight [26] and subsequently used in experiments.

### Determination of inhibitory concentration (IC_50_)

#### Preparation of resazurin

Resazurin powder (Sigma Aldrich, St. Louis, MO, USA) was dissolved in sterile distilled water at a final concentration of 0.02%, sterilized by filtration and stored at 4°C until use.

#### Preparation of the camphene thiosemicarbazide derivative

The stock solution of TSC-C was prepared in dimethyl sulfoxide (10% DMSO) and diluted to obtain the evaluated concentrations (316 μM, 158 μM, 79 μM, 39.5 μM and 19.5 μM).

The determination of IC_50_ was performed according to the micro-dilution method described in the Clinical and Laboratory Standards Institute (CLSI) [27] and De Paula et al. [28]. Were inoculated 1x10^6^ cells/mL of *P*. *lutzii* yeast cells per microplate well in MMcM liquid medium supplemented with 316 μM, 158 μM, 79 μM or 39.5 μM TSC-C. To determine the maximum growth rate (positive control), some wells received culture medium in place of the 100 mL of test compound dilution. The plates were incubated at 36°C with shaking at 150 rpm for 48 h. Each well then received 15 μL of the resazurin solution, and the plate was re-incubated for 24 h. The IC_50_ was defined as the concentration of compound capable of inhibiting 50% of cell growth of the fungus according to the absorbance at 600 nm.

### Determination of the susceptibility of *P*. *lutzii* to the camphene thiosemicarbazide derivative

The TSC-C sensitivity test was carried out on plates containing Fava-Netto semi-solid medium supplemented with TSC-C. The concentrations tested were 316 μM, 158 μM, 79 μM and 39.5 μM. Negative control plates were prepared in the absence of TSC-C. A total of 10^5^, 10^6^ and 10^7^ yeast cells were inoculated on each plate. The plates were incubated for 7 days at 36°C and photographed.

### Viability curve

Cell viability was determined using trypan blue staining and standard cell count techniques in a Neubauer chamber. We inoculated 1x10^6^ cells/mL of *P*. *lutzii* yeast cells in MMcM liquid medium supplemented with TSC-C at 79 μM—the IC_50_ concentration—for 0, 1, 2, 3, 4, 8 and 24 h of incubation. The negative control was performed in the absence of TSC-C. For counting, samples were collected at specific time points, and 10 μL of the cell solution was added to 190 μL trypan blue solution and diluted to a final volume of 1 mL. Yeast cells were observed under light microscopy with a 40X lens.

### RNA extraction and purification of mRNA

Total RNA was extracted after the incubation of *Paracoccidiodies spp*. yeast with TSC-C at 79 μM for 8 h of cultivation. The RNA was extracted with Trizol reagent (Invitrogen), precipitated with isopropanol, and resuspended with diethyl pyrocarbonate- (DEPC-) treated water. The mRNA was purified using the GenElute mRNA kit (Sigma Aldrich).

### cDNA library construction and DNA sequencing

The cDNA library was built using the SuperScript Plasmid System with Gateway Technology for cDNA Synthesis and Cloning kit (Invitrogen). The cDNA was cloned into the pCMV.SPORT6 plasmid vector and transformed into *E*. *coli* (XL1blue) cells. The cDNA library was plated at approximately 200 colonies per plate (150 mm Petri dish). The colonies were randomly selected and transferred to a 96-well polypropylene plate containing LB medium and grown overnight. Plasmid cDNA was isolated and purified.

cDNA inserts were sequenced from the 5’ end by employing standard fluorescence labeling with the DYEnamic ET dye terminator kit with an M13 flanking vector primer. Automated sequence analysis was performed in a MegaBACE 1000 DNA sequencer (GE Healthcare, Uppsala, Sweden).

### Pipeline processing and annotation of ESTs

PHRED [29], Crossmatch (http://www.macvector.com/Assembler/trimmingwithcrossmatch.html) and CAP3 [30] tools were integrated into a pipeline (http://www.lbm.icb.ufg.br/pipelineUFG/). Only sequences with at least 50 nucleotides and a PHRED quality greater or equal to 20 were considered for assembly and cluster formation. ESTs were screened for vector sequences against the UniVec data. All of the clustered sequences were queried for similarity using BLASTX (http://www.ncbi.nlm.nih.gov/BLAST) sequence comparison software against the nucleotide database generated from the *P*. *lutzii Pb*01 structural genome (http://www.broad.mit.edu/annotation/genome/paracoccidioides_brasiliensis/MulHome.html). Sequences were grouped into functional categories with the PEDANT3 database (http://pedant.helmholtz-muenchen.de/index.jsp). Similarities with E-values ≤ 10^−5^ were considered significant. The Munich Information Center for Protein Sequences (MIPS) (http://mips.gsf.de/) database was used to assign functional categories. EC numbers were obtained by the Enzyme Database-Brenda (http://www.brenda-enzymes.info)

### *In silico* determination of up-regulated genes

To assign a differential expression character, ESTs from contigs formed from yeast cells treated with TSC-C were statistically evaluated using the method by Audic and Claverie [31]. Overexpressed genes, determined by comparison to the *P*. *lutzii* transcriptome database (https://dna.biomol.unb.br/Pb/), were determined with a 95% confidence rate.

### Generation of *P*. *brasiliensis SOD1*-aRNA isolate

DNA from the *P*. *brasiliensis* wild-type strain ATCC 60855 (WT) was extracted from yeast cultures during exponential growth. We employed a high-fidelity Platinum Taq DNA polymerase (Invitrogen, Carlsbad, CA, USA) to amplify aRNA oligonucleotides designed on the PABG_03954 (www.broadinstitute.org) sequence of the *SOD1* gene. *P*. *brasiliensis* plasmid construction for aRNA and *Agrobacterium tumefaciens*-mediated transformation were performed as previously described [32]. Briefly, the amplified *SOD1*-aRNA oligonucleotides were inserted into the pCR35 plasmid under the control of the Calcium Binding Protein 1 (CBP-1) promoter region from *Histoplasma capsulatum* [33]. The pUR5750 plasmid was used as a parental binary vector to harbor the aRNA cassette within the transfer DNA (T-DNA). The constructed binary vectors were introduced into *A*. *tumefaciens* LBA1100 ultracompetent cells by electroporation as described previously [34] and isolated by kanamycin selection (100 mg/mL).

*P*. *brasiliensis* and *A*. *tumefaciens* were combined in a 1:10 ratio and incubated for 3 days of co-culture at 28°C. Selection of *P*. *brasiliensis* transformants was performed in BHI solid media containing hygromycin B (Hyg; 200 mg/mL) over a 15 day incubation period at 36°C. Randomly selected Hyg resistant transformants were tested for mitotic stability. *P*. *brasiliensis* yeast cells transformed with the empty parental vector pUR5750 (EV) were used as controls alongside the experimental yeast in the assays carried out in this study. The integration of the a-RNA cassette in the *P*. *brasiliensis* genome was confirmed by PCR analysis.

### Determination of the susceptibility of *P*. *brasiliensis* and the *SOD1*-aRNA isolate to TSC-C

To evaluate the susceptibility of *P*. *brasiliensis* to TSC-C, the WT, EV and *SOD1*-aRNA isolate strains were grown in Fava-Netto liquid medium for 72 h under constant shaking at 150 rpm and 36°C. Yeast were then transferred into MMcM liquid medium and cultured overnight. Yeast cells were then washed with 1X PBS, and the assays were performed with 1x10^6^ cells. The different isolates were distributed in solid BHI medium supplemented with 316 μM, 158 μM, 79 μM and 39.5 μM TSC-C. The controls were carried out in the same medium without the addition of TSC-C. The *SOD1*-aRNA isolated was growth in the presence of TSC-C added of ascorbic acid aiming to validate the influence of TSC-C as indutor agente of ROS. Initially, the concentrations from 0.08 to 100 mM ascorbic acid were used to determinate IC_50_ (data not shown). So, 0.2 mM ascorbic acid was added at 316 μM, 158 μM, 79 μM and 39.5 μM TSC-C. All plates were incubated for 6 days at 36°C before being photographed.

### Gene expression analysis by qRT-PCR

Total RNA was obtained from *Paracoccidioides spp*. yeast cells grown in the presence or absence of TSC-C for 8 h. After treatment with DNase, the cDNA was synthesized from total RNA using Superscript II reverse transcriptase (Invitrogen) according to the manufacturer's instructions. The primers for, ATP synthase, Superoxide dismutase (*SOD1*) [PABG_03954 (www.broadinstitute.org)], Heat shock protein 30 kDa (*HSP30)*, alcohol dehydrogenase (*ADH*), aldehyde dehydrogenase (*ALDH*) and α-tubulin genes were designed using the Primer Express software (Applied Biosystems, Foster City, CA, USA). The sequences of the oligonucleotide primers are shown in Table 1. The qRT-PCR analyses were performed in triplicate with the StepOnePlus real-time PCR system (Applied Biosystems). The expression values were calculated using the alpha tubulin transcript (XM_002796593) as the endogenous control as reported previously [35]. For transcripts of interest, relative expression levels were calculated using the standard curve method for relative quantification [36]. The relative standard curve was generated by pooling cDNAs from all conditions and serially diluting them from 1:5 to 1:625.

**Table 1:** Oligonucleotide primers used in qRT-PCR.

Sequence Name	Forward primer (5’-3’)	Reverse primer (5’-3’)	Tm (GC+AT)
**Alpha-Tubulin**	ACAGTGCTTGGGAACTATACC	GGGACATATTTGCCACTGCC	62
**Superoxide dismutase 1**	ACTGCGCAAGTTATGATGGAA	CACGGGAAGGGTCCATTTTC	62
**ATP synthase**	AAGCAGCGAAAATAATGGGATC	GCAAATAATCCTGTAGCTTCTG	62
**Heat shock protein 30 kDa**	GGCCTTGACAGCATTCTGG	CTGGCGATAAAGGGCAGAAG	62
**Alcohol dehydrogenase**	ACCTTGTTGTGCTGGAGTAGA	GGAGTCTGGAATCGGGGTG	62
**Aldehyde dehydrogenase**	CCTCTTACGGCCTTGCTGC	CGGACGCCCTTGATCTGAG	62

### Preparation of protein extracts from *P*. *lutzii*

Protein extracts were obtained after 8 h incubation in MMcM in the presence of 79 μM TSC-C or in its absence. Yeast cells were centrifuged at 10,000 x *g* for 10 min at 4°C, and the proteins were extracted using extraction buffer (20 mM Tris-HCl pH 8.8; 2 mM CaCl_2_) with a mixture of protease inhibitors (GE Healthcare). After the addition of glass beads (0.45 mm), the cells were lysed in a bead-beater, followed by centrifugation at 10,000 x *g* for 15 min at 4°C. The supernatant was collected and used in enzyme activity assays. The protein concentrations were determined using the Bradford reagent (Sigma-Aldrich), as previously described [37].

### Determination of enzymatic activity

SOD activity was measured using a commercially available kit (SOD assay Kit Sigma-Aldrich) following the manufacturer's instructions. The SOD assay kit utilizes the water-soluble tetrazolium salt-WST-1 (2-[4- Iodophenyl]-3-[4-nitrophenyl]-5-[2,4-disulfophenyl]-2H-tetrazolium, monosodium salt), which produces a water-soluble formazan dye upon reduction with a superoxide anion, and the product can be detected by a colorimetric method at 440 nm. 1 μg/mL of proteins was used in assay, and the levels of SOD activity were quantified by measuring the decrease in absorbance.

### Reactive oxygen species (ROS) detection

Intracellular H_2_O_2_ was measured by detecting the fluorescence intensity of 2`,7`-dichlorofluorescein, the oxidation product of 2`,7`-dichlorofluorescein diacetate. After treatment with 79 μM TSC-C for 4, 8 and 12 h, yeast cells were centrifuged and incubated with 20 μM 2`,7`- dichlorofluorescein diacetate for 30 min at 37°C. After washing with PBS, yeast cells were resuspended in 1 mL PBS and analyzed with a BD Accuri C6 flow cytometer (Accuri Cytometers, Ann Arbor, MI, USA). A total of 10,000 cells per sample were acquired with the FL1-H channel.

### Fluorescence microscopy

Yeast cells were inoculated in 100 mL MMcM medium at 1x10^6^ cells/mL. The cultures were incubated overnight at 36°C with gentle shaking. Cells were then centrifuged at 5,000 x *g* for 5 min and transferred into MMcM media containing 79 μM TSC-C for 4, 8 and 12 h. Control cells were incubated in MMcM without TSC-C. To detect ROS, cells were centrifuged and incubated with 20 μM 2`,7`- dichlorofluorescein diacetate for 30 min at 37°C. The specimens were analyzed with an Axio Scope A1 microscope and Axio Vison LE software (Carl Zeiss AG, Germany).

### DNA fragmentation assay

Yeast cells were treated with 79 μM TSC-C for 4, 8 and 12 h. Samples were centrifuged, the cell pellet was resuspended in 300 mL of cell lysis buffer (10 mM Tris, 0.5% Triton X-100, pH 7.5), and the sample was incubated on ice for 30 min. The lysates were centrifuged at 12,000 x *g* for 10 min at 4°C, and the supernatants were extracted once with buffered phenol and once with chloroform. DNA was precipitated with 3 M sodium acetate and butanol. DNA samples were resuspended in 50 μL Tris-EDTA buffer (10 mM Tris, 1 mM EDTA, pH 7.5) treated with RNaseA. Extracted DNA was electrophoresed through a 2% agarose gel and stained with ethidium bromide.

### Cell cycle analysis

The DNA content of yeast cells in the G0/G1, S and G2/M phases was measured using a BD Accuri C6 flow cytometer (Accuri Cytometers). Cells were incubated with 79 μM TSC-C for 4, 8 and 12 h. After treatment, the cells were collected, washed with PBS 1X, and 1x10^6^ cells/mL were fixed with cold absolute ethanol overnight at 4°C. After two washes with PBS 1X, the cells were incubated with 1 mL propidium iodide staining solution (2 μg/mL) and 50 μL RNase (10 mg/mL) and incubated for 30 min at room temperature in the dark. A total of 10,000 cells per sample were acquired with the FL2-H channel. Data were collected using FCS Express 4 Plus Research Edition software (Denovo Software, Los Angeles, CA, USA).

### Mitochondrial membrane potential measurement

The mitochondrial membrane potential was measured using rhodamine 123 (Rho123). Yeast cells were treated with 79 μM TSC-C for 4, 8 and 12 h. After treatment, the cells were collected by centrifugation and incubated with 20 μM Rho123 for 20 min at room temperature. After a PBS wash, the cells were resuspended in 1 mL PBS and analyzed using a BD Accuri C6 flow cytometer (Accuri Cytometers) with excitation and emission wavelengths of 488 and 530 nm, respectively.

### Statistical analysis

Descriptive statistics were calculated from the results, and charts were created in Microsoft Office Excel 2003 (Microsoft, Redmond, WA, USA). In this study, all of the values were expressed as arithmetic means with S.D. of triplicates. The significant differences between the groups were analyzed by Student’s t-test and *p*-values ≤0.05 were considered statistically significant.

## Results and Discussion

### The camphene thiosemicarbazide derivative affects *Paracoccidioides spp*. growth and viability

**Fig 1:**
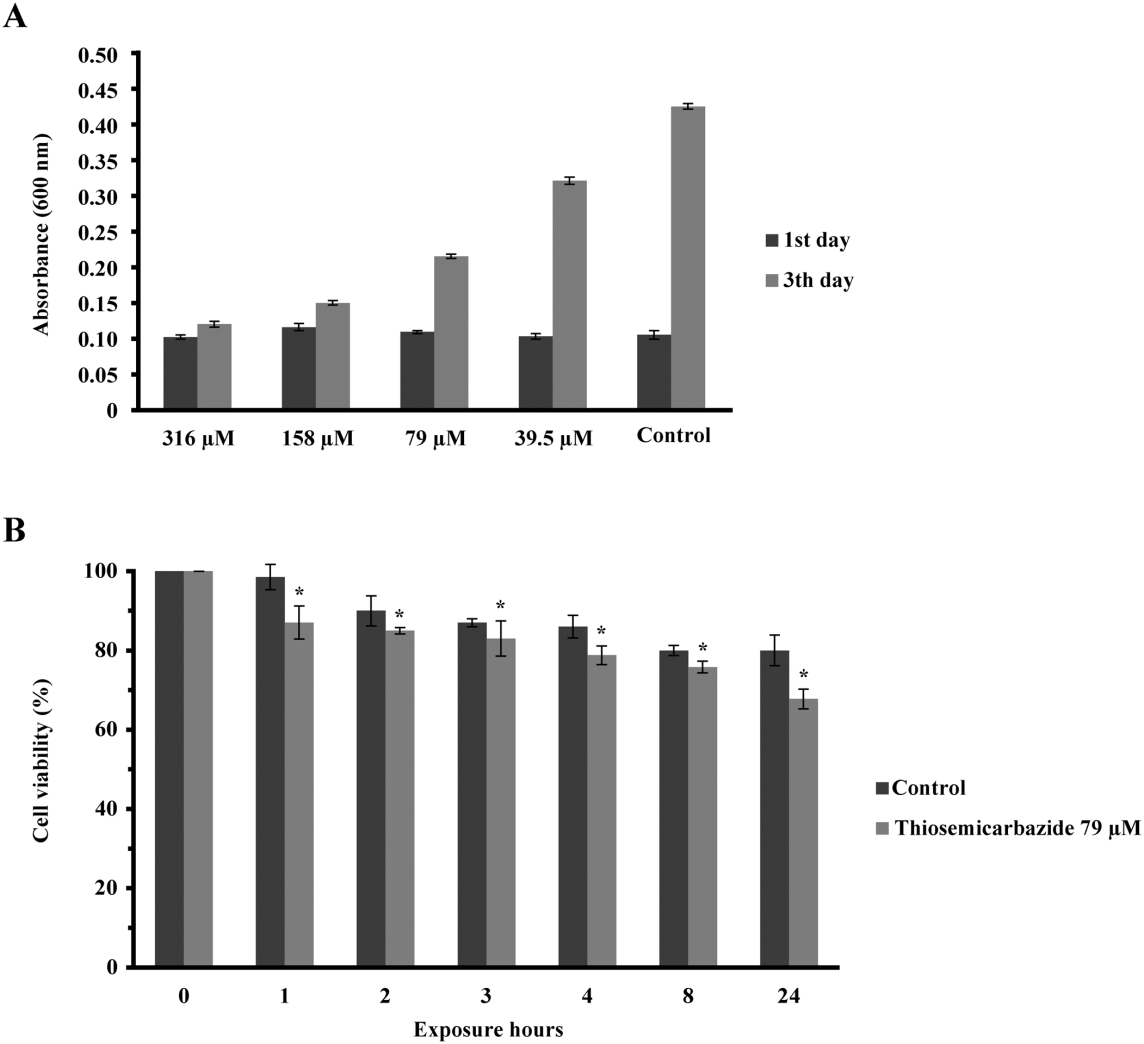
Effect of TSC-C on *P*. *lutzii* yeast cell growth. **(A)** Inhibition of *Paracoccidioide*s cell growth after treatment with TSC-C. The inhibition was visualized by addition of resazurin reagent to culture and measuring the absorbance at 600 nm. To calculate the IC_50_ value, two absorbance readings were performed; ‘1° day’ refers to reading at the beginning of the experiment, ‘3° days’ refers to reading after 3 days of incubation with 316 μM, 158 μM, 79 μM and 39.5 μM TSC-C. The positive control was performed in the absence of the compound. **(B**) Cell viability after 1, 2, 3, 4, 8 and 24 h exposure to TSC-C. The data are presented as percentage of cell viability. The Student’s *t*-test was used for statistical comparisons, and the observed differences were statistically significant (*p* ≤ 0.05). The error bars represent the standard deviation of three biological replicates.

**Fig 2:**
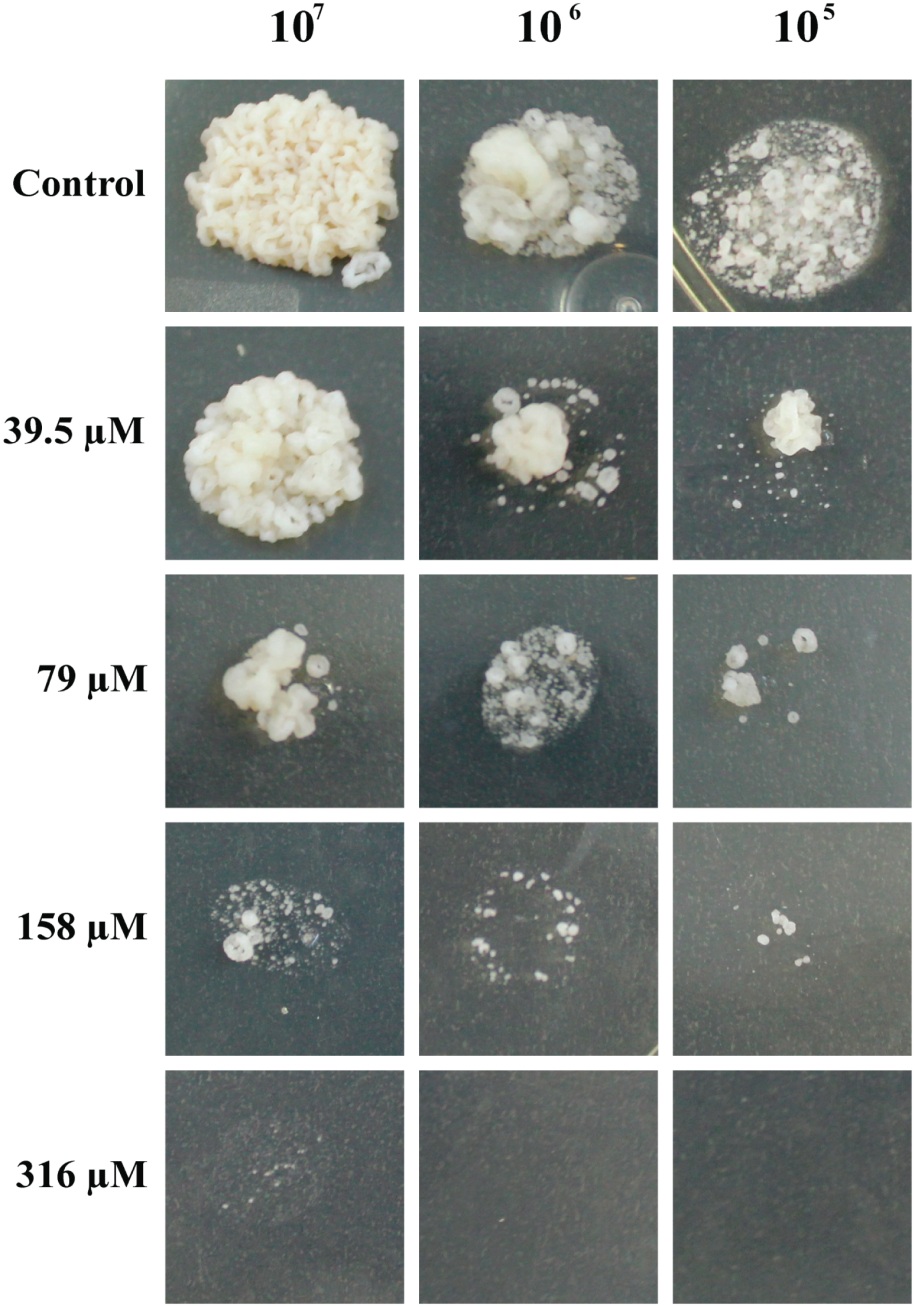
Susceptibility of *P*. *lutzii* yeast cells exposed to TSC-C. Samples containing 1x10^7^, 1x10^6^ and 1x10^5^ yeast cells were spotted on Fava-Netto plates supplemented with TSC-C at the concentrations indicated above. The plates were incubated for 7 days at 36°C before photo documentation.

Here, we aimed to evaluate the effect of TSC-C on *P*. *lutzii*. The cells were incubated in the presence of TSC-C. Fig 1A demonstrates that TSC-C inhibited yeast growth in a dose-dependent manner. TSC-C at a concentration of 79 μM inhibited the cellular growth by 50% and became the IC_50_ value of TSC-C for *Paracoccidioides* yeast. Additionally, the cellular viability of the fungus was monitored in the presence of 79 μM TSC-C for 24 h. Fig 1B reveals that the yeast cell viability drops to 85% after 8 h of exposure to TSC-C, time used for the transcriptomic analysis. The dose-dependent inhibition was also observed in yeast cells grown on the solid medium supplemented with different concentrations of TSC-C (Fig 2). TSC-C (79 μM) was not toxic to Balb 3T3 cells (data not shown), These results confirmed the antifungal activity of this compound.

### cDNA library construction and overview of ESTs from *P*. *lutzii* exposed to TSC-C

A cDNA library was constructed to determine the expression profile of *Paracoccidioides spp*. exposed to TSC-C. The dosage and duration of antifungal treatment are known to be critical steps in adaptive gene expression [38]; thus, the choice of these parameters was necessary for the construction of a cDNA library. The concentration used in the experiments was 79 μM corresponding to IC_50_ of TSC-C for *P*. *lutzii*. The fungus was exposed to TSC-C for 8 h, since exhibited 85% viability.

We obtained a total of 2,012 clones, and 1,844 of these were successfully sequenced. All sequences were arranged into 68 contigs and 686 singlets representing different transcripts. Of these, 33 genes were down-regulated and 84 genes were up-regulated when compared to the transcriptome *P*. *lutzii* yeast cells grown *in vitro*. A total of 64 genes were unique to TSC-C-treated *P*. *lutzii* yeast cells. The ESTs obtained were submitted to the National Center for Biotechnology Information (NCBI) under accession numbers: LIBEST_028508 *Paracoccidioides* thiosemicarbazide Library.

### Functional annotation and analysis of sequences

All up- and down-regulated ESTs were compared to *Paracoccidioides Pb*01 genes in the Broad Institute database with the Blast X program. Only ESTs with e-value < 10^−5^ were considered in this analysis. All contigs and singlets were annotated with Blas2GO. The ESTs were grouped according to the MIPS functional annotation scheme (Munich Information Center for Protein Sequences) into general functional categories affected by TSC-C. The ESTs were related to metabolism; cell cycle and DNA processing; biogenesis of cellular components; cellular communication/signal transduction mechanism; cell rescue, defense and virulence; energy; protein synthesis; protein fate; translation; and unclassified proteins (Table 2).

**Table 2:** Functional classification of up and down-regulated genes from *P*. *lutzii* yeast cells in the presence TSC-C.

Functional classification/ Accession number	Gene product	EC number	Number of occurences ESTs
**Metabolism**			
***Amino acid metabolism***			
**PAAG_00468.2**	4-aminobutyrate aminotransferase	2.6.1.19	+3
**PAAG_04052.2**	**Homoserine O-acetyltransferase**	2.3.1.31	+2
***C-compound and carbohydrate metabolism***			
**PAAG_00771.2**	Enolase	4.2.1.11	-1
**PAAG_05580.2**	**NAD dependent epimerase/dehydratase family protein**	5.1.3.2	+2
**PAAG_08949.2**	**GPI Mannosyltransferase**	2.4.1	+2
***Lipid*, *fatty acid and isoprenoid metabolism***			
**PAAG_05837.2**	Palmitoyl-protein thioesterase	3.1.2.22	+2
**PAAG_06329.2**	3-hydroxybutyryl-CoA dehydrogenase	1.1.1.157	+1
**PAAG_06215.2**	**Hydroxymethylglutaryl-coa lyase**	4.1.3.4	+5
**PAAG_08410.2**	Acyl-coenzyme A:6-aminopenicillanic-acid-acyltransferase 40 kDa form	2.3.1.164	+2
**PAAG_03203.2**	Protoheme IX farnesyltransferase	2.5.1	+2
***Nitrogen*, *sulfur and selenium metabolism***			
**PAAG_00954.2**	Urease	3.5.1.5	+2
***Nucleotide/nucleoside/nucleobase metabolism***			
**PAAG_04291.2**	Nucleoside diphosphate kinase	2.7.4.6	-5
**Cell cycle and DNA processing**			
***DNA recombination and DNA repair***			
**PAAG_04357.2**	**DNA mismatch repair protein**		+2
**PAAG_05988.2**	**DNA-repair protein rad2**		+2
**PAAG_04646.2**	**Mold-specific protein MS95**		+2
***Cell cycle***			
**PAAG_02186.2**	**Nuclear segregation protein Bfr1**		+2
**PAAG_00513.2**	**Cell division control protein**		+2
**PAAG_07814.2**	**Subunit of condensin complex**		+2
**PAAG_03188.2**	**Nuclear movement protein NUDC**		+2
**Biogenesis of cellular componentes**			
***Cytoskeleton/structural proteins***			
**PAAG_05855.2**	**Ankyrin repeat domain containing protein**		+2
**Cellular communication/signal transduction mechanism**			
***Cellular signalling***			
**PAAG_03783.2**	GAF domain nucleotide-binding protein		+3
**PAAG_03386.2**	cAMP—Dependent protein kinase catalytic subunit	2.7.11.11	+3
**PAAG_03923.2**	**TRAF-type zinc finger protein**		+2
**PAAG_01861.2**	Membrane associated progesterone receptor component 1	1.6.2.2	+5
**Cell rescue, defense and virulence**			
***Stress response***			
**PAAG_00871.2**	Heat shock protein 30 kDa		-1
**PAAG_04164.2**	Superoxide dismutase	1.15.1.1	+5
**Cellular transport, transport facilities and transport routes**			
***Transport routes***			
**PAAG_00782.2**	**Small COPII coat GTPase sar**		+2
**PAAG_07328.2**	**Transport protein SEC61 subunit alpha**		+4
**PAAG_09049.2**	**ENTH domain-containing protein**		+2
**PAAG_05643.2**	Endoplasmic reticulum and nuclear membrane proteinc Npl4		-3
**PAAG_08587.2**	GPR1/FUN34/yaaH family protein		-1
**PAAG_05251.2**	High affinity copper transporter		-1
***Transported compounds (substrates)***			
**PAAG_00635.2**	**ABC transporter CDR4**	3.6.3.44	+3
**Energy**			
***Glycolysis and gluconeogenesis***			
**PAAG_00403.2**	Alcohol dehydrogenase	1.1.1.1	-4
***Energy conversion and regeneration***			
**PAAG_04570.2**	ATP synthase D chain, mitochondrial	3.6.3.14	-2
***Respiration***			
**PAAG_08901.2**	**Glyoxylate reductase**	1.1.1.26	+2
**PAAG_05249.2**	Aldehyde dehydrogenase	1.2.1.3	-2
***Electron transport and membrane-associated energy conservation***			
**PAAG_06595.2**	3-demethylubiquinone-9 3-methyltransferase		+5
**PAAG_05031.2**	**NADH-ubiquinone oxidoreductase 40 kDa subunit**	1.6.5.3	+3
**PAAG_01307.2**	**NADH dehydrogenase iron-sulfur protein**	1.6.99.3	+2
**PAAG_07593.2**	Cytochrome-c oxidase chain VIIc	1.9.3.1	+2
**Protein synthesis**			
***Ribosome biogenesis***			
**PAAG_09043.2**	Ribossomal protein 40S—S2		-1
**PAAG_01785.2**	Ribossomal protein 40S—S3		-1
**PAAG_05017.2**	Ribossomal protein 40S—S10-A		-3
**PAAG_01433.2**	Ribossomal protein 40S—S14		-1
**PAAG_00088.2**	Ribossomal protein 60S—L3		-2
**PAAG_07955.2**	Ribossomal protein 60S—L18		-1
**PAAG_00205.2**	Ribossomal protein 60S—L24		-1
**PAAG_04965.2**	Ribossomal protein 60S—L31		-5
**PAAG_07841.2**	Ribossomal protein 60s—P1		-2
**PAAG_03664.2**	Ribossomal protein L28e		-2
**PAAG_00206.2**	Ribossomal protein S30		-2
**PAAG_07649.2**	Ribossomal protein S36		-2
**PAAG_03828.2**	Ribossomal protein 40 S-S9		-1
***Translation***			
**PAAG_02024.2**	Elongation factor 1-alpha		-2
**PAAG_07105.2**	**Isoleucyl-tRNA synthetase**	6.1.1.5	+2
**Protein fate (folding, modification, destination)**			
***Assembly of protein complexes***			
**PAAG_05879.2**	Complex I intermediate-associated protein		-2
**PAAG_02594.2**	**Phosphoprotein phosphatase 2C**	3.1.3.16	+2
***Protein folding and stabilization***			
**PAAG_05788.2**	**Peptidyl-prolyl cis-trans isomerase A2**	5.2.1.8	+2
***Protein/peptide degradation***			
**PAAG_05052.2**	**AAA Family ATPase**		+2
***Protein modification***			
**PAAG_05777.2**	**Dual specificity phosphatase catalytic domain containing protein**		+2
**Transcription**			
***RNA synthesis***			
**PAAG_07098.2**	Histone H4.1		+8
**PAAG_08917.2**	Histone H2a		+5
**PAAG_08532.2**	**Ribonuclease Z**	3.1.26.11	+2
**PAAG_00891.2**	**AT hook motif Family protein**		+2
**Unclassified Proteins**			
**PAAG_04823.2**	Hypothetical protein		+3
**PAAG_03567.2**	**Hypothetical protein**		+6
**PAAG_05112.2**	**Hypothetical protein**		+2
**PAAG_04156.2**	**Hypothetical protein**		+2
**PAAG_01567.2**	**Hypothetical protein**		+3
**PAAG_03475.2**	**Hypothetical protein**		+2
**PAAG_03684.2**	**Hypothetical protein**		+4
**PAAG_03129.2**	**Hypothetical protein**		+3
**PAAG_06864.2**	**Hypothetical protein**		+2
**PAAG_01497.2**	**Hypothetical protein**		+2
**PAAG_07875.2**	**Hypothetical protein**		+5
**PAAG_04268.2**	Hypothetical protein		+3
**PAAG_02546.2**	Hypothetical protein		+4
**PAAG_08699.2**	**Hypothetical protein**		+3
**PAAG_04869.2**	**Hypothetical protein**		+4
**PAAG_02037.2**	**Hypothetical protein**		+2
**PAAG_02676.2**	**Hypothetical protein**		+3
**PAAG_07947.2**	**Hypothetical protein**		+3
**PAAG_08808.2**	**Hypothetical protein**		+2
**PAAG_00149.2**	**Hypothetical protein**		+2
**PAAG_01940.2**	**Hypothetical protein**		+3
**PAAG_07462.2**	**Hypothetical protein**		+3
**PAAG_01216.2**	**Hypothetical protein**		+2
**PAAG_02607.2**	Hypothetical protein		+3
**PAAG_05412.2**	**Hypothetical protein**		+2
**PAAG_05607.2**	**Hypothetical protein**		+4
**PAAG_05415.2**	**Hypothetical protein**		+2
**PAAG_07420.2**	**Hypothetical protein**		+2
**PAAG_07885.2**	**Hypothetical protein**		+3
**PAAG_00947.2**	**Hypothetical protein**		+2
**PAAG_02407.2**	**Hypothetical protein**		+2
**PAAG_08549.2**	**Hypothetical protein**		+3
**PAAG_08355.2**	**Hypothetical protein**		+2
**PAAG_02237.2**	**Hypothetical protein**		+2
**PAAG_02996.2**	Hypothetical protein		+18
**PAAG_07710.2**	Hypothetical protein		+3
**PAAG_04455.2**	**Hypothetical protein**		+4
**PAAG_06704.2**	**Hypothetical protein**		+2
**PAAG_08976.2**	**Hypothetical protein**		+2
**PAAG_04152.2**	**Hypothetical protein**		+2
**PAAG_05097.2**	**Hypothetical protein**		+2
**PAAG_06142.2**	**Hypothetical protein**		+2
**PAAG_01456.2**	**Hypothetical protein**		+2
**PAAG_04691.2**	Hypothetical protein		-2
**PAAG_04913.2**	Hypothetical protein		-1
**PAAG_04707.2**	Hypothetical protein		-2
**PAAG_03385.2**	Hypothetical protein		-5
**PAAG_07334.2**	Hypothetical protein		-4
**PAAG_04431.2**	Hypothetical protein		-7
**PAAG_08722.2**	Hypothetical protein		-2
**PAAG_00415.2**	Hypothetical protein		-3
**PAAG_03232.2**	Hypothetical protein		-2

Genes in bold correspond to single genes in condition TSC-C. The signs + and - represent induced and repressed genes, respectively.

**Fig 3:**
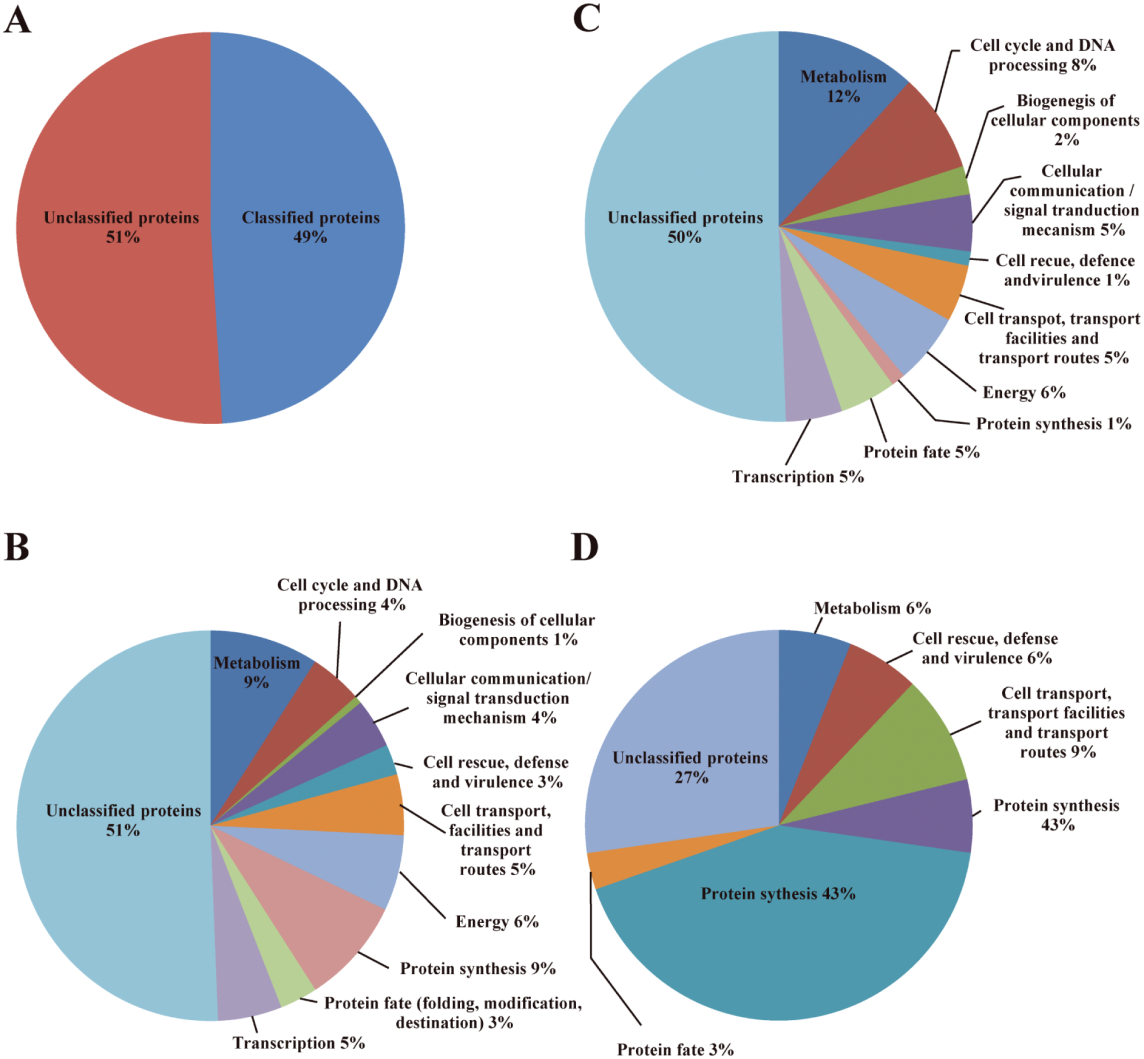
Statistically enriched MIPS functions. **(A)** Total ESTs represented by classified and unclassified categories. **(B)** Genes expressed differentially in the presence of camphene thiosemicarbazide derivate. Up- **(C)** or down- **(D)** regulated *P*. *lutzii* genes after exposure of yeast cells to TSC-C. The functional classification was based on the MIPS functional annotation scheme. Each functional class is represented as a color-coded segment and expressed as a percentage of the total number of ESTs.

Graphs were plotted to demonstrate the statistically enriched MIPS functions with up- or down-regulated genes after exposure to the compound. A total of 51% (161 ESTs) were associated with proteins of unknown function (Fig 3A). Transcriptome analysis revealed that ESTs associated with metabolism (9%) and protein synthesis (9%) were the most highly represented after 8 h of TSC-C exposure (Fig 3B). The TSC-C treatment resulted in the up- and down-regulation of genes involved in different biological processes (Table 2; Fig 3C and 3D). The groups with the highest percentage of up-regulated genes were unclassified proteins (50%); metabolism (12%); cell cycle and DNA processing (8%); energy (6%); transcription (5%); protein fate (5%); cellular transport, transport facilities and transport routes (5%); biogenesis of cellular components (2%); protein synthesis (1%); and cell rescue, defense and virulence (1%) (Fig 3C). The highest percentage of down-regulated genes were grouped within protein synthesis (43%); unclassified proteins (27%); cellular transport, transport facilities and transport routes (9%); energy (6%); and cell rescue, defense and virulence (3%) (Fig 3D).

We analyzed transcript occurrence by determining the number of ESTs found for each transcript. The transcripts with the highest occurrence of up-regulated ESTs were as follows: hypothetical protein PAAG_02996 (18 ESTs), histone H 4.1 (8 ESTs), hypothetical protein PAAG_03567 (6 ESTs), hypothetical protein PAAG_07875 (5 ESTs), histone H2a (5 ESTs), membrane-associated progesterone receptor component 1 (5 ESTs), 3-demethylquinone-9 3-methyltransferase (5 ESTs), hydroxymethylglutaryl-CoA lyase (5 ESTs) and superoxide dismutase (5 ESTs). For down-regulated ESTs, the highest abundance were as follows: hypothetical protein PAAG_04431 (7 ESTs), hypothetical protein PAAG_03385 (5 ESTs), nucleoside diphosphate kinase (5 ESTs) and ribosomal protein 60S –L31 (5 ESTs).

### Description of transcripts changed during exposure to TSC-C

ABC transporter *CDR4* was induced in TSC-C-treated *P*. *lutzii* yeast cells. These are transmembrane proteins that utilize energy generated by the hydrolysis of adenosine triphosphate (ATP) to carry out biological processes including the translocation of various substrates across membranes [39]. In addition, they are involved in multidrug resistance in other human pathogens such as *Candida albicans* [40,41] *Aspergillus fumigatus* [42,43] and *Cryptococcus neoformans* [44]. Notably, its induction has been correlated with the protection of *Aspergillus nidulans* against cytotoxic agents [45].

Similarly, most genes related to protein fate were induced in the presence of TSC-C. Conversely, genes related to protein synthesis, mainly ribosomal proteins, were inhibited. It is well established that ribonuclease inhibitors such as the vanadyl ribonucleoside complex (VRC) can inhibit RNases involved in ribosomal subunit formation, resulting in a decreased rate of ribosomal subunit synthesis [46].

Another gene strongly repressed in the presence of TSC-C was the endoplasmic reticulum and nuclear membrane protein *NPL4*. In *Saccharomyces cerevisiae*, the Npl4p protein is part of a highly conserved protein complex required for the proteasome-mediated processing and activation of ER-membrane-bound transcription factors, resulting in proper membrane fluidity and organelle function. Furthermore, the perturbation of membrane composition in mutant npl4 cells leads to the loss of ER/nuclear envelope integrity, which in turn causes the observed defects in nuclear transport [47].

Here, we observed the down-regulation of a high affinity copper transporter (Table 2), suggesting that TSC-C could interfere with copper homeostasis on *Paracoccidioides spp*. Copper is an essential cofactor for a wide variety of enzymes in crucial biological processes critical for cell growth, differentiation and survival [48,49]. Some studies suggest that copper acquisition plays an important role in the virulence of *C*. *neoformans* [50]. However, at high intracellular concentrations, copper can be toxic due to the perturbation of the cellular redox potential, which increases production of reactive free radicals and indirectly increases oxidative stress [51]. In *S*. *cerevisiae*, the high-affinity copper transport proteins, which play an important role in regulating copper homeostasis, are induced by copper deprivation and repressed by copper excess [52,53].

**Fig 4:**
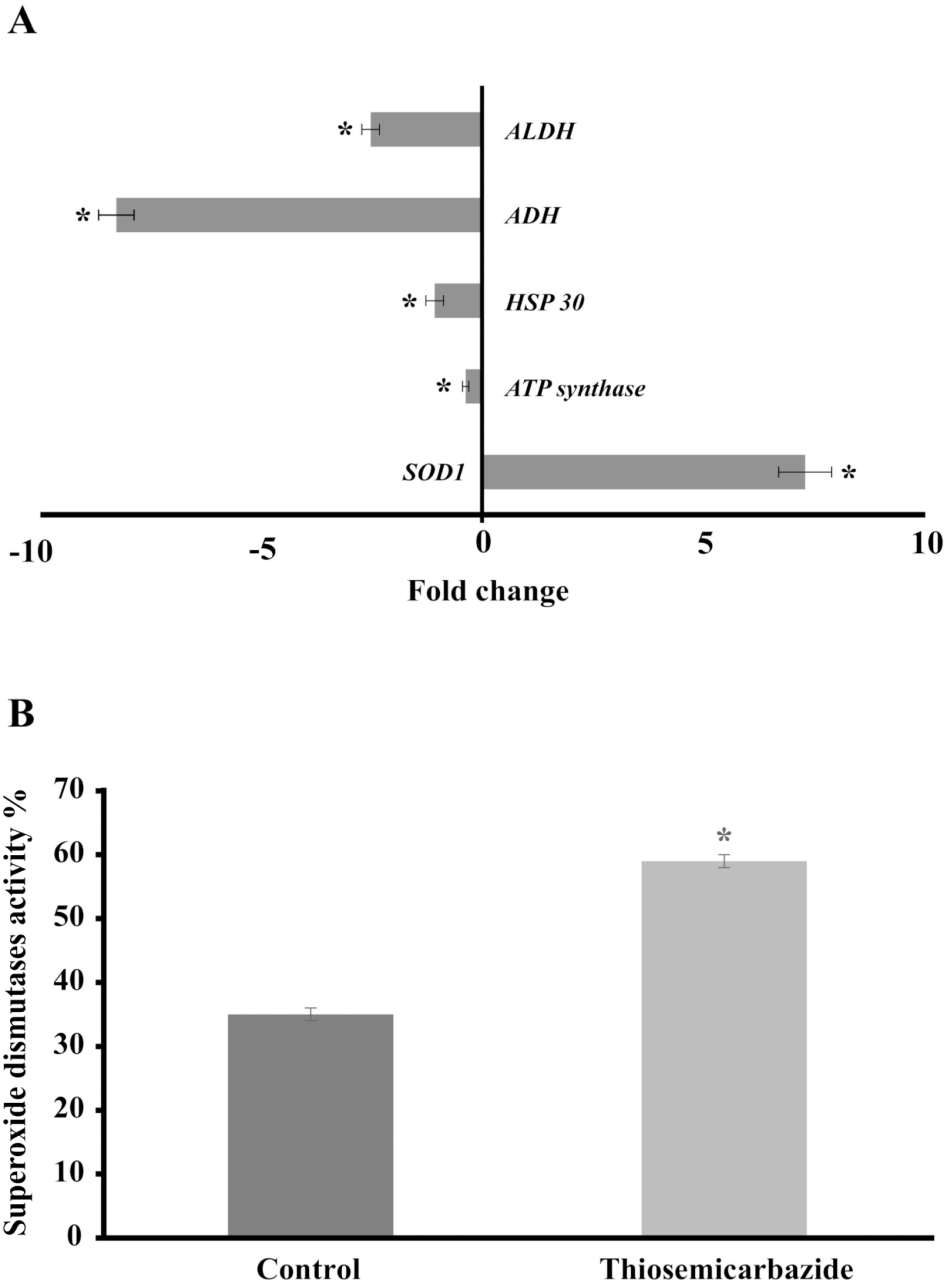
Effect of TSC-C on the genes and *SOD1* activity of *P*. *lutzii* **(A)** Gene expression profile of yeast cells exposed to TSC-C for 8 h. Changes in the gene expression levels were calculated by the relative standard curve method using the non-treated control samples to calibrate. Each error bar represents the standard error of the mean (±SD), and significant fold changes are denoted by asterisks in the figure (*p≤0.05). Data were normalized with the transcript encoding the α-tubulin protein. **(B)** SOD1 activity. Yeast cells were grown in the presence of TSC-C for 8 h, and total protein was extracted to measure SOD1 activity. The Student’s *t*-test was used for statistical comparisons, and the observed differences were statistically significant (*p*≤0.05). Error bars represent the standard deviation of three biological replicates.

The mitochondrial electron transport chain performs the transfer of electrons from glycolysis and the Krebs cycle and thereby creates an electrochemical gradient and energy, which is used for a variety of vital processes that include, ATP synthesis [54], ion homeostasis [55], protein import [56] and programmed cell death [57]. Because energy metabolism and redox state are potential targets, the development of drugs that specifically compromise the structural and functional integrity of mitochondria may provide novel opportunities to combat fungal infections [58]. Studies *in vitro* have demonstrated the interaction between drugs and mitochondria that may prove useful in several therapies [59,60,61]. In this sense, mitochondrial insult or failure can rapidly lead to the inhibition of cell survival and proliferation [62]. Furthermore, here we uncovered that genes related to electron transport, such as NADH-ubiquinone oxidoreductase 40 kDa subunit, NADH dehydrogenase iron-sulfur protein and cytochrome-c oxidase chain VII c, were induced in the presence of TSC-C; however, the expression of the ATP synthase D chain was inhibited (Fig 4A), suggesting that TSC-C could destabilize the electron transport chain and, as a consequence, decrease the production of ATP in *P*. *lutzii*.

### TSC-C induces *SOD1* up-regulation as a consequence of TSC-C-induced ROS

Superoxide dismutases (SODs) constitute the primary antioxidant defense against ROS, promoting dismutation of the superoxide radical (O^2−^) into molecular oxygen and H_2_O_2_, which are less toxic for the cell. ROS-generating agents induce fungal SODs, among other proteins [63]. *SOD3* protects *H*. *capsulatum* yeast cells from host-derived oxidative stress by detoxifying ROS produced by macrophages and neutrophils, thereby enabling the survival of the fungus [64]. In the case of *Paracoccidioides spp*., the induction of *SOD1* protein is similarly observed in cells exposed to H_2_O_2_ [65]. In *C*. *albicans*, another fungal pathogen, the inactivation of ROS detoxifying enzymes has been shown to attenuate its virulence [66].

**Fig 5:**
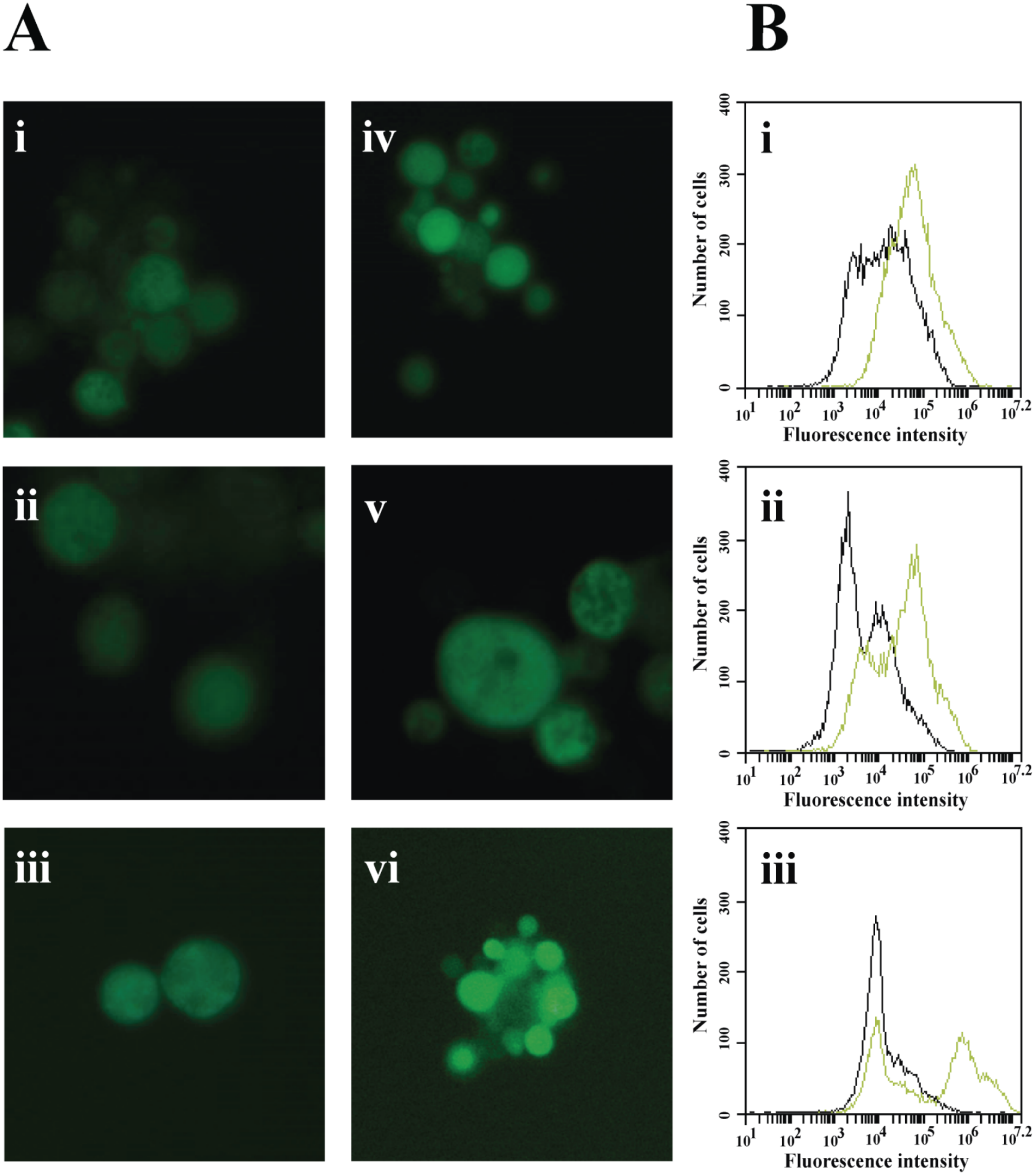
Formation of ROS by TSC-C. (A) Fluorescence microscopy of *P*. *lutzii* yeast cells stained with 2`,7`-dichlorofluorescein diacetate. Yeast cells were grown in the absence of TSC-C for **i)** 4 h, **ii)** 8 h and **iii)** 12 h and in the presence of TSC-C for **iv)** 4 h, **v)** 8 h and **vi)** 12 h. **(B)** Flow cytometry analysis of yeast cells grown in the absence or in the presence of TSC-C. The cells were monitored for **i)** 4 h, **ii)** 8 h and **iii)** 12 h stained with 2`,7`-dichlorofluorescein diacetate. Black histograms represent control yeast cells, and green histograms represent yeast cells treated with TSC-C.

**Fig 6:**
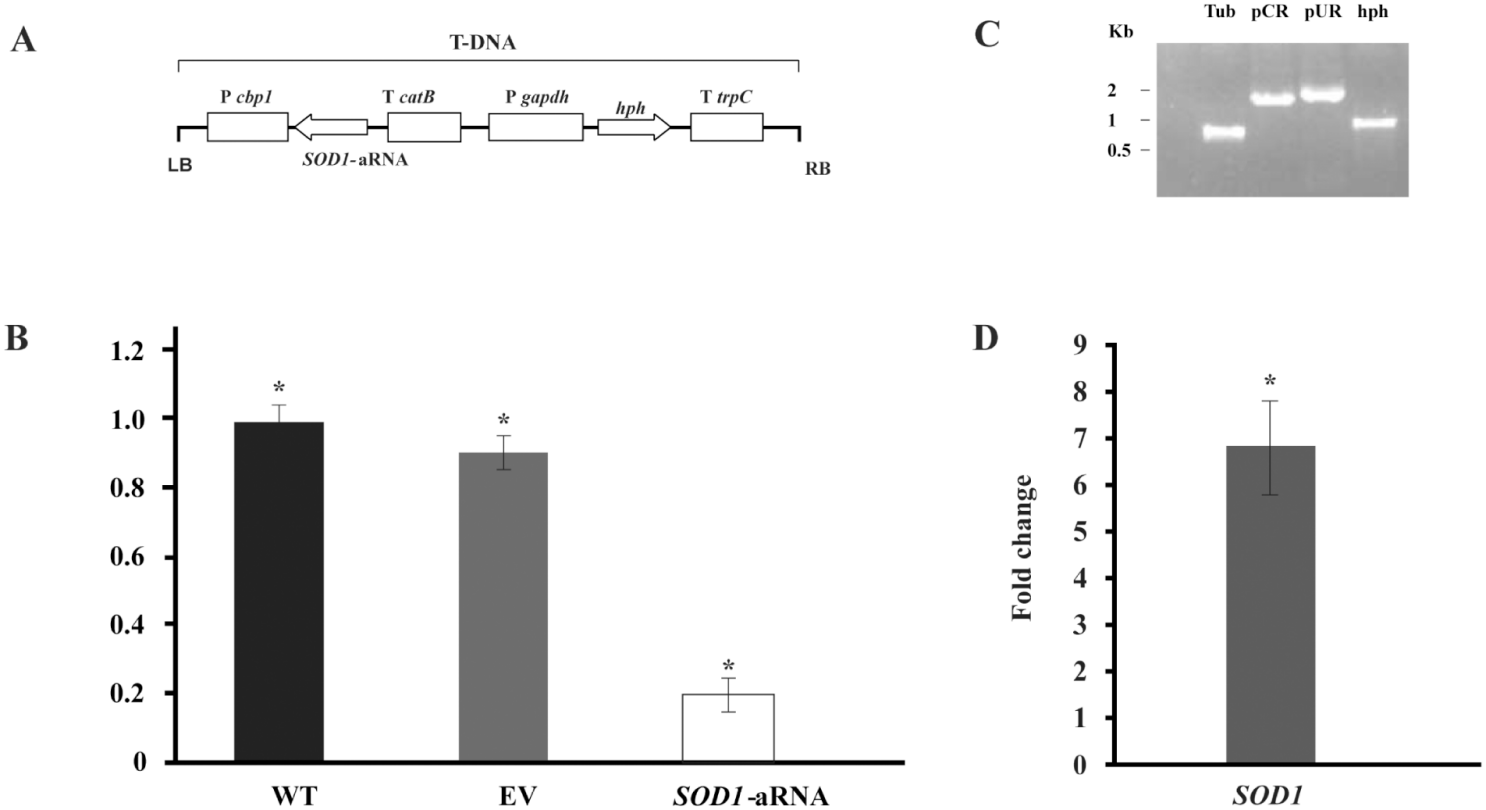
Gene silencing of *SOD1* in *P*. *brasiliensis*. **(A)** Transfer DNA (T-DNA) inserted into the genome of *P*. *brasiliensis* yeast cells via ATMT in order to silence the *SOD1* gene. The antisense oligonucleotide was directed to exon 3 (black box) that amplify a length of 85 bp. This AS oligonucleotide was placed under the control of the calcium binding protein (*CBP*-1) with a terminator (*CAT*-B); the plasmid contained hygromycin B phosphotransferase (*HPH*) under the control of glyceraldehyde 3-phosphate of *Aspergillus nidulans* (*PGPDA*) with a terminator (*TTRCP*). **(B)** *PbSOD1* gene expression levels obtained by RT-qPCR. The measurement was normalized with the housekeeping gene alpha-tubulin in WT, EV and *SOD1*-aRNA yeast cells growing in the exponential phase. Mitotic stability was confirmed by sub-culturing *P*. *brasiliensis SOD1*-aRNA yeast cells and testing for low expression levels in this isolate after successive sub-cultures. (C) Validation by PCR of the presence and integration of the Transfer DNA (T-DNA) into the genome of *P*. *brasiliensis* transformant. The genomic DNA from the *SOD1*-aRNA isolate was tested by PCR using specific primers for the alpha-tubulin gene *TUB* (Tub, lane 3), for the transformation constructs pCR35 (pCR, lane 4) and pUR5750 (pUR, lane 5) and for the hygromycin resistance gene (hph, lane 6). (D) *SOD1* expression profile in *P*. *brasiliensis* after exposure to TSC-C. RNA was extracted after 8 h of exposure of yeast cells to TSC-C. Changes in gene expression levels were calculated by the relative standard curve method using the non-treated control samples to calibrate.

Here, we confirmed the up-regulation of *SOD1* in *P*. *lutzii* and *P*. *brasiliensis* by qRT-PCR (Figs 4A and 6D) and by enzymatic activity assay in *P*. *lutzii* (Fig 4B), suggesting that TSC-C could induce the formation of ROS leading to oxidative stress in *Paracoccidioides spp*. yeast cells. Thus, to confirm our hypothesis, we evaluated the production of ROS by means of fluorescence microscopy and flow cytometry. In yeast cells treated with TSC-C, we observed an increase in fluorescence in a time-dependent manner, indicating that this compound could be inducing the formation of ROS in *P*. *lutzii* (Fig 5A and 5B). The main classes of antifungal drugs used in the treatment of invasive fungal infections, such as azoles, polyenes and echinocandins, are also capable of inducing ROS production [67,68].

**Fig 7:**
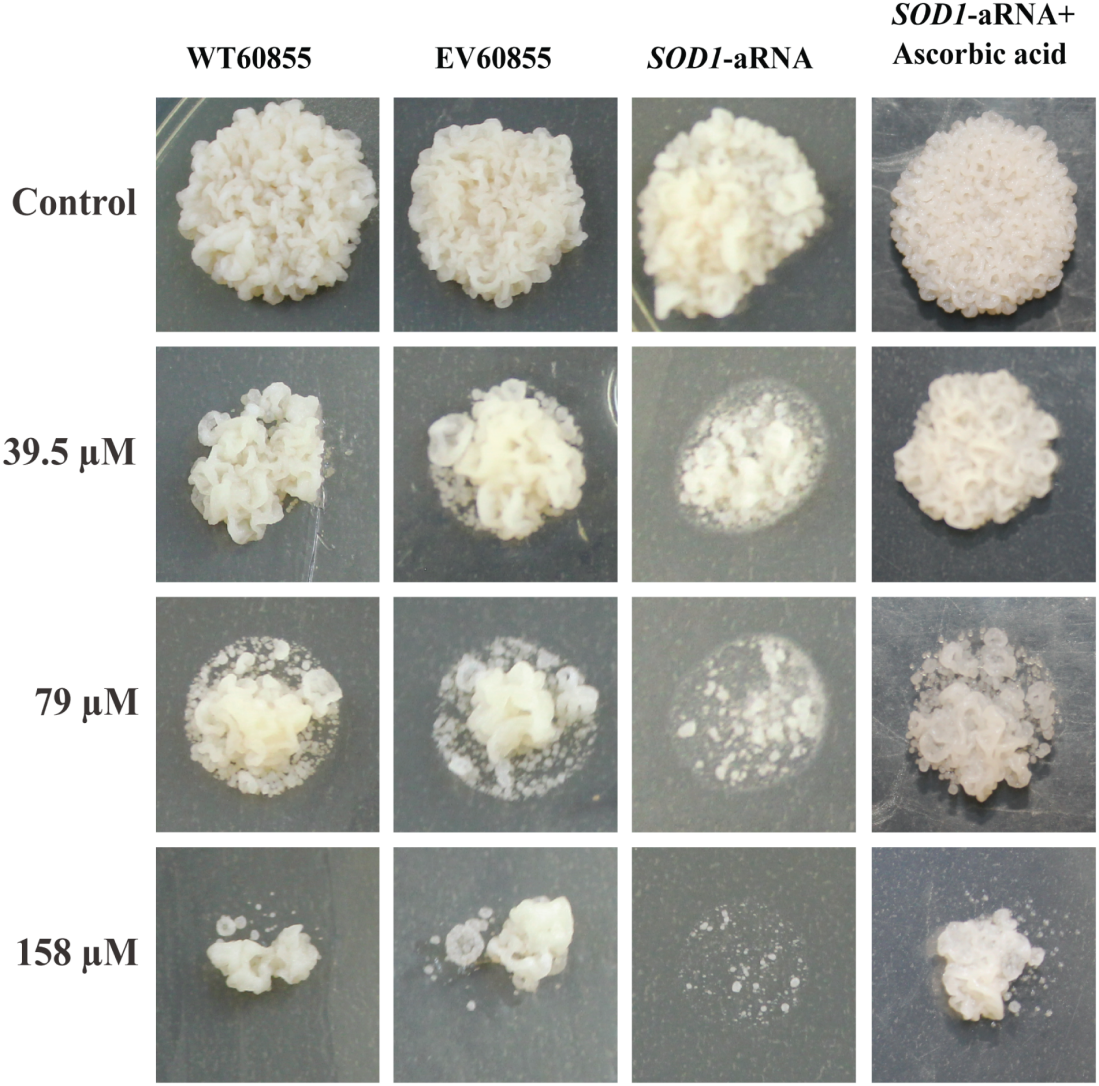
Susceptibility of *P*. *brasiliensis SOD1*-aRNA to TSC-C. 1x10^6^ yeast cells of *P*. *brasiliensis* WT60855, EV60855 and *SOD1*-aRNA were spotted on solid BHI supplemented with 39.5, 79 and 158 μM TSC-C. Control cells were spotted on BHI without TSC-C or with 39.5, 79 and 158 μM TSC-C and ascorbic acid. The plates were incubated for 7 days at 36°C before photo documentation.

Taking into account the above results, we inquired about the importance of *SOD1* during TSC-C treatment. To validate our assumption, we created a *P*. *brasiliensis* isolate (*SOD1*-aRNA) with down-regulated *SOD1* gene expression (Fig 6A and 6B). The PCR analysis was performed to confirm the integration of the a-RNA cassette in the *P*. *brasiliensis* genome (Fig 6C). *SOD1*-aRNA was obtained to ATCC 60855 isolate since *SOD1* was also induced in this isolate (Fig 6D) and the protocol to obtainment of mutant to *P*. *brasiliensis* has not yet standardized. The susceptibility of ATCC60855 and *SOD1*-aRNA to TSC-C was evaluated (Fig 7). When *SOD1*-aRNA isolate cells were exposed to TSC-C, we observed a reduced growth rate relative to WT and EV yeast cells. The growth was restored in the presence of antioxidant, ascorbic acid. This result corroborates our transcriptional data that indicate an up-regulation of *SOD1* during TSC-C treatment and suggests that the up-regulation of this gene is important for *Paracoccidioides* survival in the presence of the compound.

### TSC-C-induced ROS leads to the collapse of the *P*. *lutzii* mitochondrial membrane

**Fig 8:**
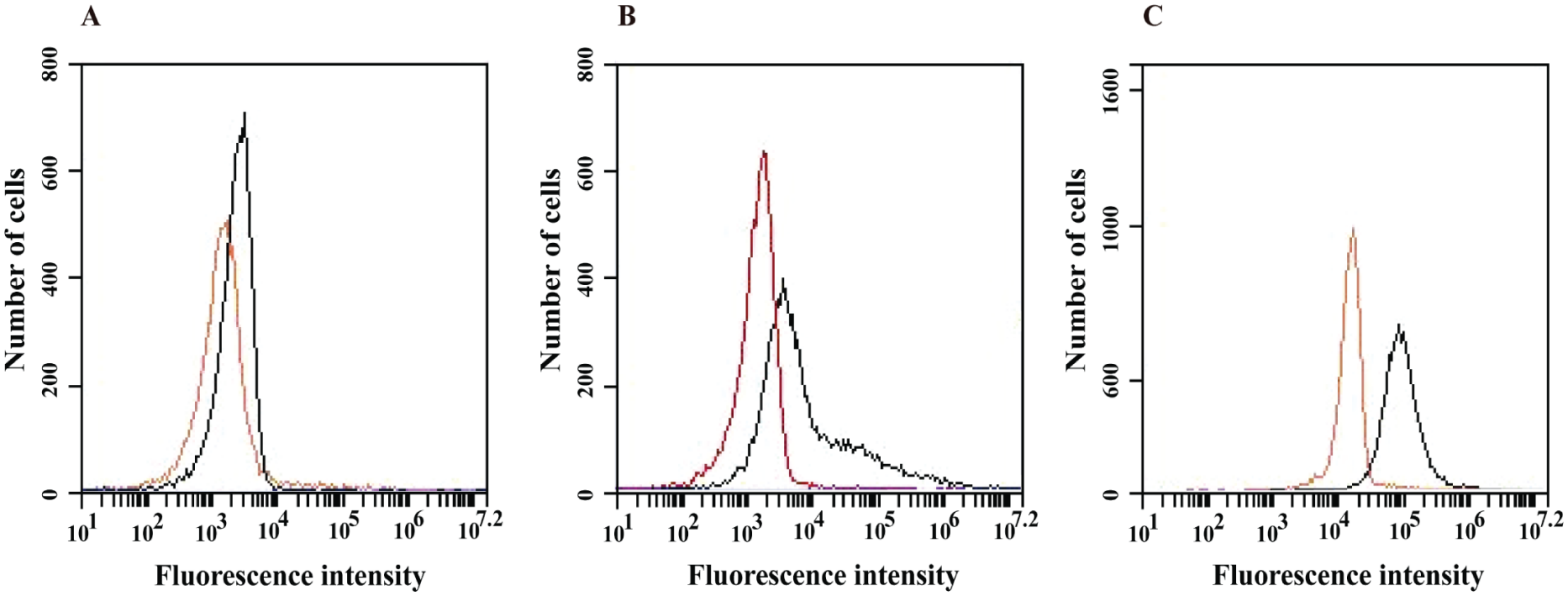
Effect of TSC-C on the mitochondrial membrane potential of *P*. *lutzii*. The mitochondrial membrane potential (ΔΨm) was determined by flow cytometry analysis of yeast cells treated with TSC-C for **A)** 4 h, **B)** 8 h and **C)** 12 h and stained with rodhamine123. Histograms in black represent the controls, and red histograms represent cells treated with TSC-C.

Mitochondrial ROS production can lead to the oxidative damage of mitochondrial proteins, membranes and DNA, thereby impairing the ability of this organelle to synthesize ATP and carry out its wide range of metabolic functions; such functions include the tricarboxylic acid cycle, fatty acid oxidation, the urea cycle and amino acid metabolism, which are pivotal for the normal function of most cells [69]. Furthermore, ROS also would cause a transition in mitochondrial permeability. This transition consists of the loss of the mitochondrial membrane potential, resulting from the formation of pores, and subsequent cell death [70]. Because TSC-C induces the production of ROS, we evaluated the mitochondrial membrane integrity by estimating the electric potential (ΔΨm) with fluorescence in yeast grown in the presence of TSC-C for 4, 8 and 12 h. Flow cytometry analysis revealed a ΔΨm decrease in the yeast cells exposed to the compound relative to control cells (Fig 8), suggesting that the TSC-C-induced ROS lead to the collapse of the mitochondrial membrane.

### TSC-C inhibits cell proliferation by changing the expression profile of genes related to the cell cycle

Cell cycle and DNA processing were among the major classes of overexpressed genes in *P*. *lutzii* cells exposed to TSC-C. From these, 8 were unique to yeast cells grown in the presence of TSC-C, and these included DNA mismatch repair protein and DNA repair protein *RAD2*. Therefore, we explored the possibility that the induction of these genes was associated with DNA damage through a DNA fragmentation assay. In fact, we did not observe DNA fragmentation in the samples cultured in the presence of TSC-C for any of the times tested (S1 Fig).

**Fig 9:**
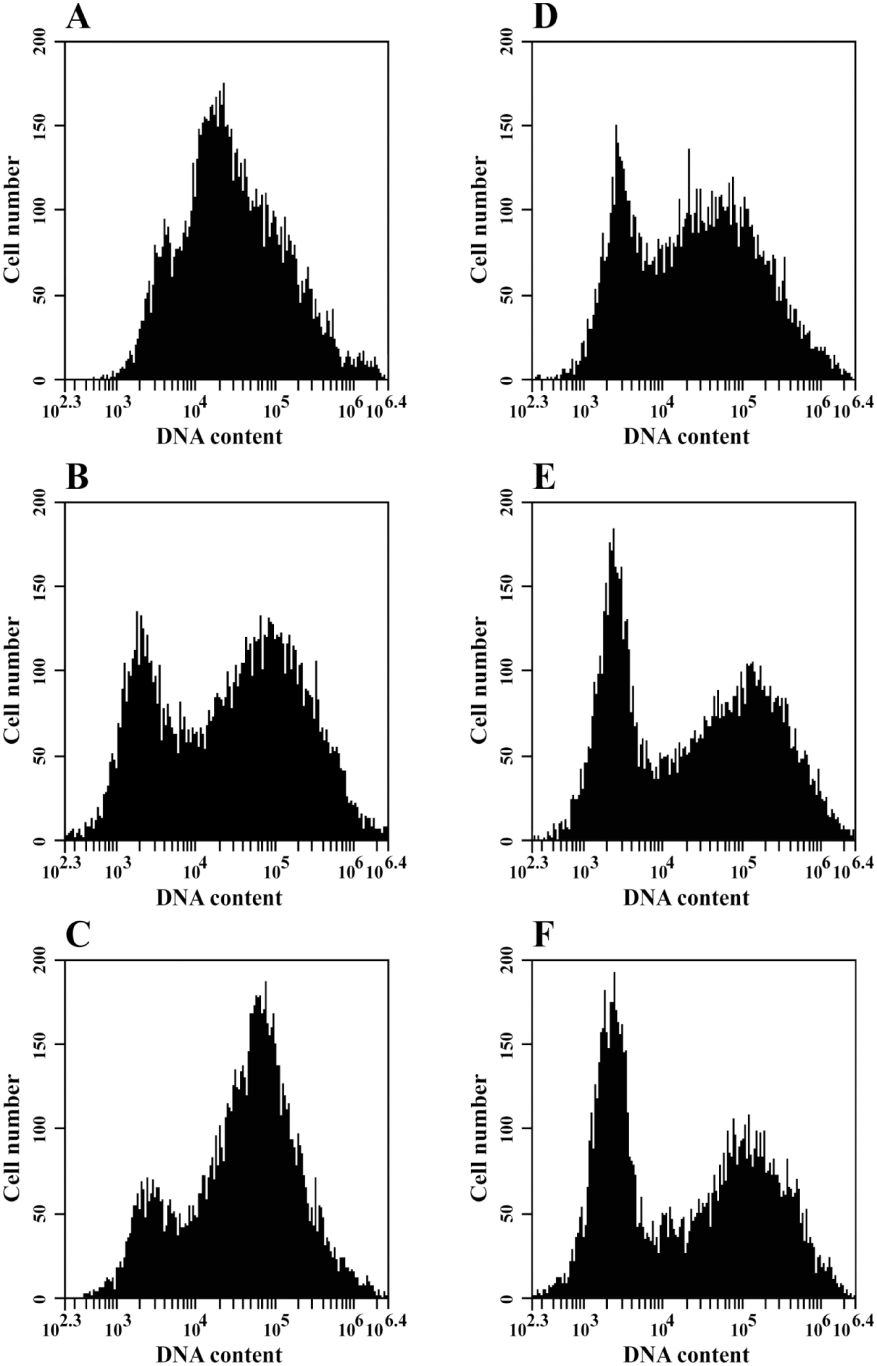
Effect of TSC-C on the *P*. *lutzii* cell cycle. The DNA content of yeast in each cell cycle phase was analyzed by flow cytometry in the absence of TSC-C for **A)** 4 h, **B)** 8 h and **C)** 12 h or in the presence of TSC-C for **D)** 4 h, **E)** 8 h and **F)** 12 h and subsequently stained with ethidium iodide as represented by histograms.

Considering the identity of the genes differentially regulated by the presence of the compound, we evaluated the *P*. *lutzii* cell cycle by analyzing DNA content by flow cytometry. The phase of the cell cycle was determined by the difference in DNA content between cells in the pre-replicative (G0 and G1) phases, the replicative (S) phase (DNA synthesis) and the post-replicative plus mitotic (G2+M) phases [71]. The results showed that the percentage of yeast cells in the G1 phase increased in a time-dependent manner after exposure to TSC-C; furthermore, the number of cells in the S and G2 phases decreased (Fig 9). Altogether, these results indicate that TSC-C inhibits cell proliferation by changing the expression profile of genes related to the cell cycle.

## Conclusion

TSC-C seems to induce the formation of ROS in *Paracoccidioides spp*., leading to the collapse of the mitochondrial membrane, and also to inhibit cell proliferation by changing the expression of genes related to the cell cycle. Relevant genes related to protein synthesis, copper homeostasis and cellular response induced by drugs or stress conditions were also observed in *Paracoccidioides* yeast cells exposed to TSC-C. The high percentage of unclassified proteins found here indicates that further studies are needed in order to better understand how TSC-C affects *Paracoccidioides spp*.

## Supporting Information

S1 Fig DNA fragmentation assay. DNA fragmentation was carried out in P. lutzii yeast cells exposed to TSC-C at 79 μM for 4, 8 and 12 h. The controls were performed with yeast cells incubated in the absence of TSC-C.
(TIF)
