# Supplementary figures and images for: Transcriptome Profile of the Response of Paracoccidioides spp. to a Camphene Thiosemicarbazide Derivative
Source: PLoS One. 2015 Jun 26;10(6):e0130703. doi: 10.1371/journal.pone.0130703 (PMC4483234; doi:10.1371/journal.pone.0130703)

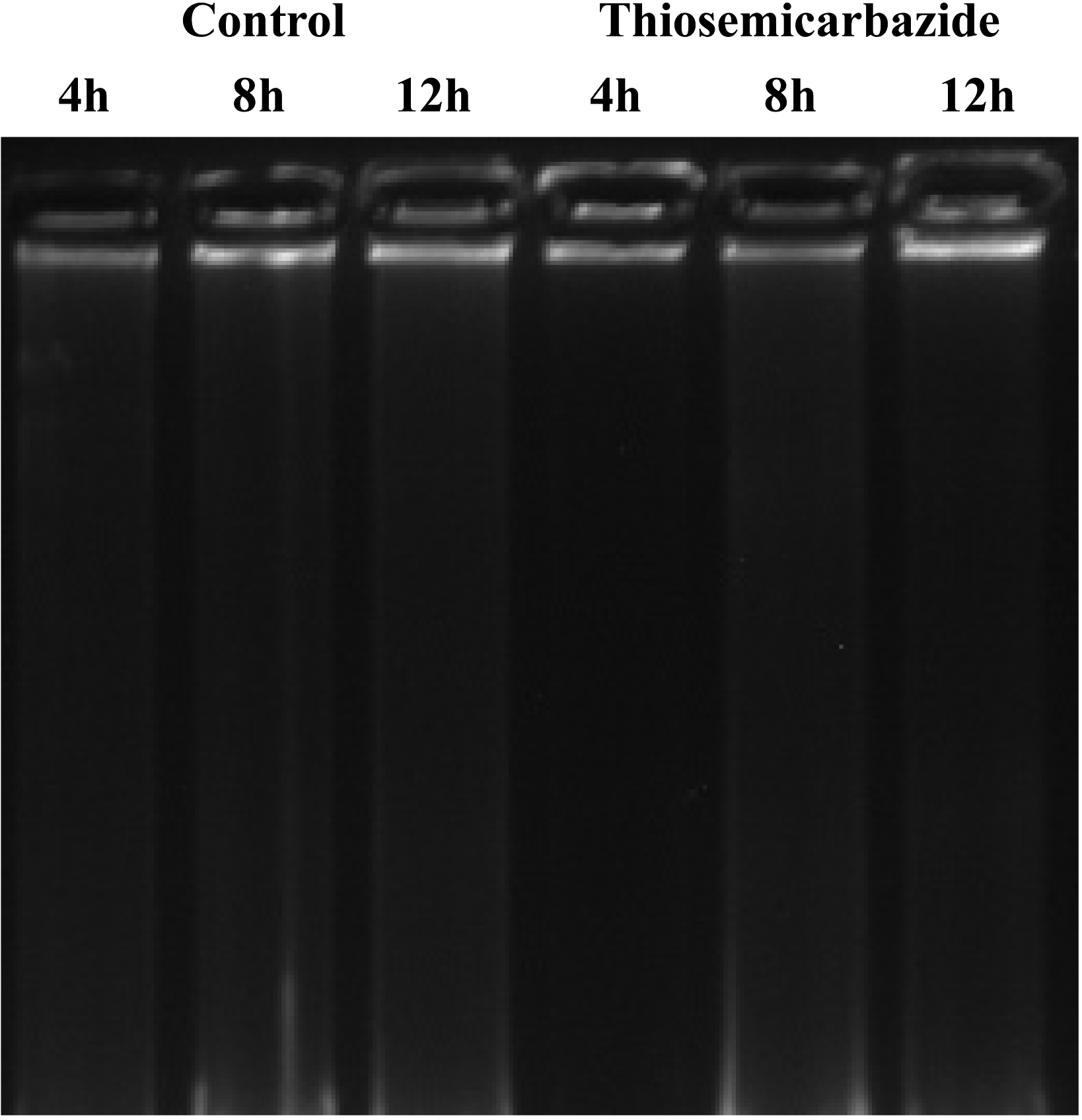

Supplement: S1 Fig — DNA fragmentation was carried out in P. lutzii yeast cells exposed to TSC-C at 79 μM for 4, 8 and 12 h. The controls were performed with yeast cells incubated in the absence of TSC-C. (TIF) [file pone.0130703.s001.tif]
